# Supplementary material for: Anopheles albimanus is a Potential Alphavirus Vector in the Americas
Source: Am J Trop Med Hyg. 2022 Dec 19;108(2):412–23. doi: 10.4269/ajtmh.22-0417 (PMC9896319; doi:10.4269/ajtmh.22-0417)
Supplement: Supplementary file 1 [file tpmd220417.SD1.pdf]

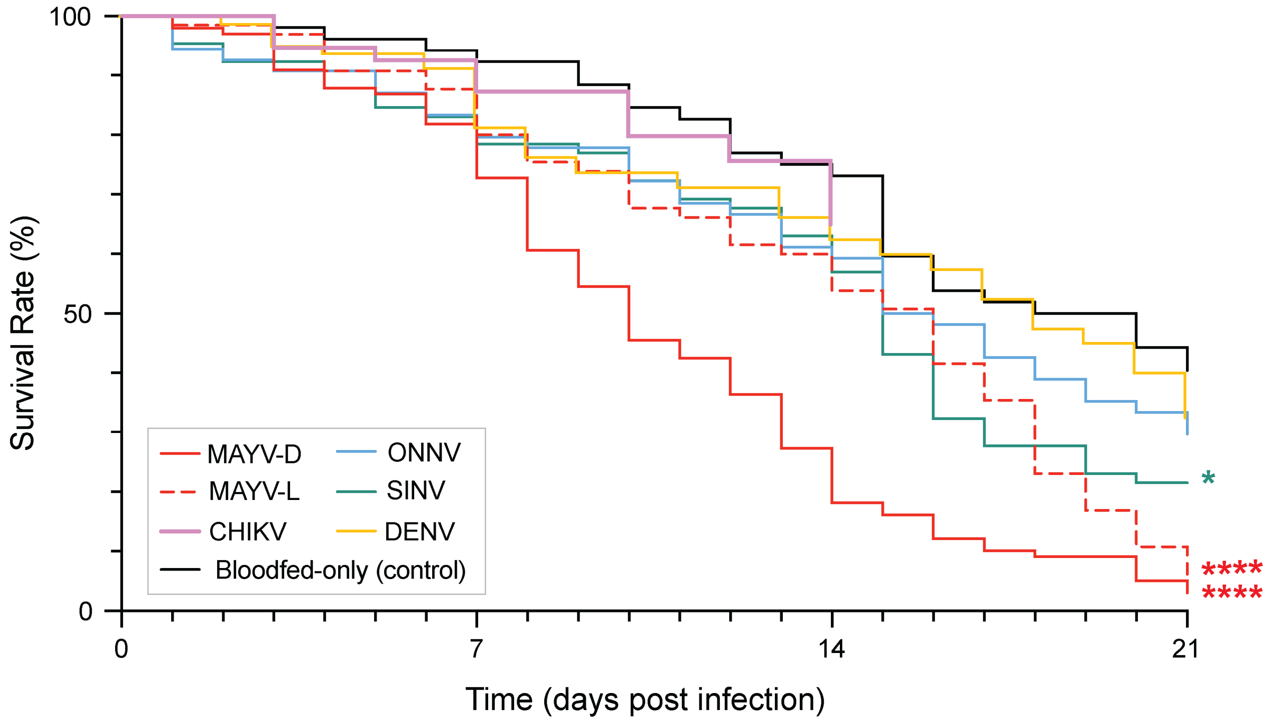

Figure S1. – *An. albimanus'* mortality associated to challenge and infection with the different viruses. Statistical significance between virus-treated and bloodfed-only samples is indicated by stars (**** p<0.0001; * p<0.05) and performed by curve comparison using a survival log-rank Mantel-Cox test.

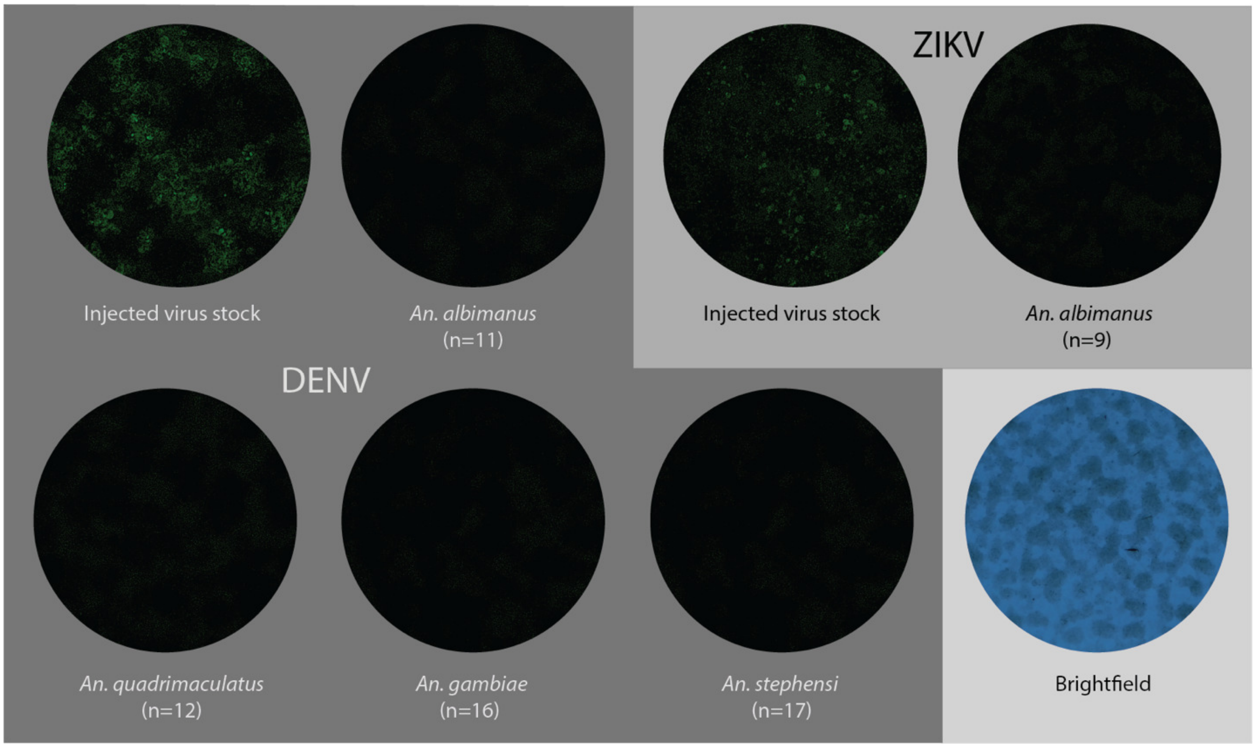

Figure S2. – Four diverse *Anopheles* species were injected intrathoracically with DENV and ZIKV to assess for presence of a flavivirus-specific midgut barrier in the genus. The figure depicts FFA on samples of intrathoracically-injected mosquitoes. FFAs on viral stocks serve as positive controls for both techniques and infective virus at time of injection, with each virus stained in green using a specific primary antibody coupled with an Alexa Fluor 488 secondary antibody.
